# Unused and expired medicines waste in Ireland – an audit of medicines returned to and generated in community pharmacies

**DOI:** 10.1007/s11845-026-04436-6

**Published:** 2026-05-13

**Authors:** Aisling C. O’Leary, Zlata Gerasimova, Neil Devlin, Arun Katheri, John Syjo, Stephen James Walsh, Matthew Lynch

**Affiliations:** School of Pharmacy and Biomolecular Sciences, RCSI University of Medicine and Health Sciences, St Stephens Green, Dublin, Ireland

**Keywords:** Environmental sustainability, Unused medicines, Medication waste, Sustainability

## Abstract

**Background:**

Medication waste is defined as *‘any pharmaceutical product that remains unused or is not fully consumed during the entire pharmaceutical supply chain’*. Given the growing recognition of the environmental impact of healthcare waste, an understanding of the scale and sources of medicines waste generated in Ireland is required.

**Aim:**

This study aims to quantify the amount of patient-returned medicines and pharmacy-generated medication waste in a sample of community pharmacies in Ireland, and to explore underlying causes and potential economic impacts.

**Methods:**

A medicines waste audit was conducted in four community pharmacies in April/May 2025. The audit tool captured data on the type, form, quantity, ATC code, source and given reason for return or wastage. The value of the waste was calculated based on the wholesaler list price.

**Results:**

The majority of wasted medicines was from patient returns (72%), and prescription medicines accounted for 90% of all wasted medicines. Patient death (21%), treatment discontinuation (13%) and non-adherence (13%) were the primary reasons for patient-returned medicines. Amendments to blister-packs (31%), expired medicines (27%) and uncollected medicines (16%) were the main drivers of pharmacy-generated waste. The mean value of medication waste per pharmacy was €1,512 over four weeks or €19,662 annually, extrapolating to a national estimate of €37.5 million.

**Conclusion:**

There is a clear need for targeted interventions to minimise medication waste across the medicines use phases involving key stakeholders. Moving towards a more sustainable healthcare system would not only lessen environmental harm, but also promote more efficient resource use and improve patient care.

## Introduction

Environmental sustainability has become an urgent global priority, as human activity continues to accelerate ecological degradation and threaten planetary health [1]. The healthcare sector, a significant contributor to environmental pollutants, must reduce its impact, but doing so is inherently complex as it must balance environmental responsibility with the delivery of safe, effective, and equitable patient care [2, 3]. Medicines, an essential component of modern healthcare, play a vital role in maintaining and restoring health, yet their production, use, distribution and disposal generate significant environmental pollutants [4]. Medicine consumption has increased over recent decades, driven by the rising prevalence of chronic and age-related conditions, as well as evolving prescribing practices, clinical guidelines, and rising patient-driven medicines’ consumption [5]. In a recent report it was estimated, that patient use of medicines grew by 14% over the past five years, and is expected to grow by a further 12%, equivalent to 400 billion defined daily doses up to the end of 2028 [6].

Medicines, comprising of active pharmaceutical ingredients (APIs) and excipient materials as well as their pharmaceutical intermediates and metabolites, can enter the environment at multiple stages of their lifecycle, including manufacture, use, and disposal. Improper disposal practices such as discarding medicines down sinks or sanitary systems allow pharmaceutical residues to reach wastewater systems and terrestrial sites [7–10]. Conventional wastewater treatment plants are generally unable to fully remove these contaminants, allowing pharmaceuticals to persist in treated effluent [11]. Similarly, medicines discarded in municipal waste contribute to pharmaceutical compounds in landfill leachate, representing another pathway of environmental contamination [12].

As a result, pharmaceuticals are now widespread environmental contaminants, with residues of commonly used medicines detected in aquatic systems worldwide, including Ireland [13–16]. Even at low concentrations, these substances can disrupt ecosystems and pose risks to wildlife [9, 17–19]. Although individual pharmaceutical compounds are generally present at concentrations below those associated with acute risk to human health, the broader effects of these compounds and their metabolites are not fully understood [10, 20]. The presence of antibiotic agents and their residues in the environment is particularly problematic, as it contributes to the emergence and spread of antibiotic-resistant bacteria, an urgent global health threat [21–23]. In addition, there are risks associated with the stockpiling of unused medicines, for whatever reason, in the home setting [24].

Medication waste is defined as any pharmaceutical product that remains unused or is not fully consumed during the entire pharmaceutical supply chain [25]. According to global estimates, between 3 and 50% of medications remain unused with around 40% being preventable [26]. The manufacture, distribution and disposal of these products contribute substantially to greenhouse gas (GHG) emissions [27, 28], making unused medicines an avoidable source of environmental burden. Medication waste also represents a misuse of financial resources for healthcare that could otherwise have been allocated to more effective patient care [29].

It is estimated that approximately 29,000 kg of medicines remain unused or expire annually in Ireland [30], yet no publicly funded national scheme for the collection and disposal of medicines is yet operational (April 2026), nor is there clear guidance available for patients on what to do with unused or expired medicines. Instead, an informal discretionary waste collection service has been provided by community pharmacies, with responsibility falling on individual retail pharmacy businesses to manage and finance disposal through private waste contractors [31]. This arrangement has potentially led to lower medicine return rates compared with other OECD countries [32], as pharmacies are understandably reluctant to encourage the large-scale return to them of waste medicines, whose disposal they must exclusively fund [33]. A survey among the Irish public conducted in 2010 and 2011 reported that unused medicines were kept in the home by 88% of respondents and of those who had disposed of medicines in the past, 72% had done so inappropriately [34]. In 2023, AlMutairi et al. drew attention to the environmental and public health risks associated with informal medicine return systems and called for a systems-based approach to address the issue of household unused and expired medicines [35]. In 2021, the EPA National Hazardous Waste Management Plan 2021–2027 recommended having a national collection system for surplus or out of date medicines by the end of 2023, which is, as yet, not established [30]. However, progress has recently been made with a change to the community pharmacy contract announced in September 2025, that from 2026 community pharmacies, will receive funding to support them to safely dispose of waste medicines returned to them [36].

Internationally, formal audits of medicines waste in community pharmacies have been conducted in several European countries and in Australia and New Zealand [37–43]. Given the growing recognition of the environmental impact of healthcare waste, there is a compelling need to better understand the scale and sources of medicines waste in Ireland. To our knowledge, this study represents the first formal audit in Ireland focusing on medication waste generated in community pharmacies.

## Aim of study

This study aims to systematically characterise and quantify medication waste generated in a sample of community pharmacies in Ireland over a defined period, and to explore the underlying causes and potential economic impacts. The objectives were:

- To quantify the amount of patient-returned medicines and pharmacy-generated medication waste.
- To identify the primary therapeutic classes, stated reasons contributing to medication waste and estimated value of the waste generated.

## Method

### Study design

A clinical waste audit was conducted in four community pharmacies located in suburban areas of Dublin, Ireland. Participating pharmacies were those where students were completing their eight-month practice placement in fulfilment of their Year 5 Master of Pharmacy programme requirements. The students’ preceptors agreed to their students conducting the audit in their pharmacies, prior to the commencement of the audit. The study was conceptualised and supervised by Dr. Aisling O’Leary and Dr. Matthew Lynch.

The audit was conducted over a four-week period, from 14th April to 11th May 2025. Each pharmacy routinely accepted unwanted, unused or expired medicines returned by patients as part of their standard operating procedures. Pharmacy staff in each participating pharmacy were informed of the study aims and objectives and given training on processes around accepting returned medicines from patients.

### Audit tool development

An audit tool was collaboratively developed by the research team of supervisors and students, to capture data on medication waste. Medicines waste generated was categorised based on its source: (1) patient-returned unused or expired medicines, and (2) pharmacy-generated medication waste including waste arising from routine pharmacy operations or expired while in the pharmacy. The audit tool was informed by the Royal Pharmaceutical Society’s audit guide for wasted medicines [44]. For each medicine, the following information was documented: (1) medication brand name, (2) prescription or over-the-counter (OTC), (3) form, (4) strength, (5) quantity, (6) unit cost price, (7) Anatomical Therapeutic Chemical (ATC) classification code, and (8) given reason for return or wastage.

### Data collection

Data collection occurred on all working days over the four-week audit period. No exclusion criteria were applied during data collection, and all products presented as waste were recorded. Exclusions were applied during data collation. Data were collected and recorded on-site using the standardised audit tool.

Patients returning medicines were asked to provide a reason for return which was recorded by staff, who also recorded reasons for pharmacy-generated medication waste. Participating staff assisted by collecting returned and wasted medicines during the working day and placing them in a designated collection area.

### Data analysis

Data from the four pharmacies were collated into a structured Microsoft Excel^®^ spreadsheet and reviewed for consistency, completeness and accuracy. Excluded items included medical devices and accessories (e.g., ostomy products, unused diabetic test strips and lancets), nutritional drinks, products without active pharmaceutical ingredients (no ATC code), and homoeopathic preparations. Products that could not be verified as originating from the Irish market through standard wholesalers were also excluded from the study due to the inability to assign a reliable economic value.

The cost of the waste medication was estimated using wholesaler list prices, without factoring in any purchasing discounts provided to the pharmacies. When a product was available in multiple commonly used pack sizes, the largest marketed pack was used for reference. For creams, ointments, and liquid preparations, the full pack size was recorded rather than estimating the remaining quantity.

The value of all waste generated in the four pharmacies during the audit period was calculated and, an average value generated per pharmacy for a four-week period was estimated and extrapolated to an annual estimate of medicines waste per pharmacy. Estimates of the postulated total value of medicines waste in Ireland were then derived, based on the total number of registered community pharmacies.

## Results

### Characteristics of medicines waste

Over the four-week audit period, 311 individual wasted medicines were recorded across the four pharmacies, of which 299 (96.1%) were included in the final analysis. Twelve medicines matching the exclusion criteria were excluded. The majority of wasted medicines were attributable to patient-returned medicines (72%), while 28% were categorised as pharmacy-generated (Table 1).

**Table 1:** Total quantity of medicines waste stratified by source

Source	Number of items	Total no. of units	% of total items
Patient-returned	216	6451	72%
Pharmacy-generated	83	2035	28%
Total	**299**	**8486**	**100%**

Of the total medication waste recorded, 90% were prescription medicines and 10% were OTC products.

### ATC code category

**Fig. 1:**
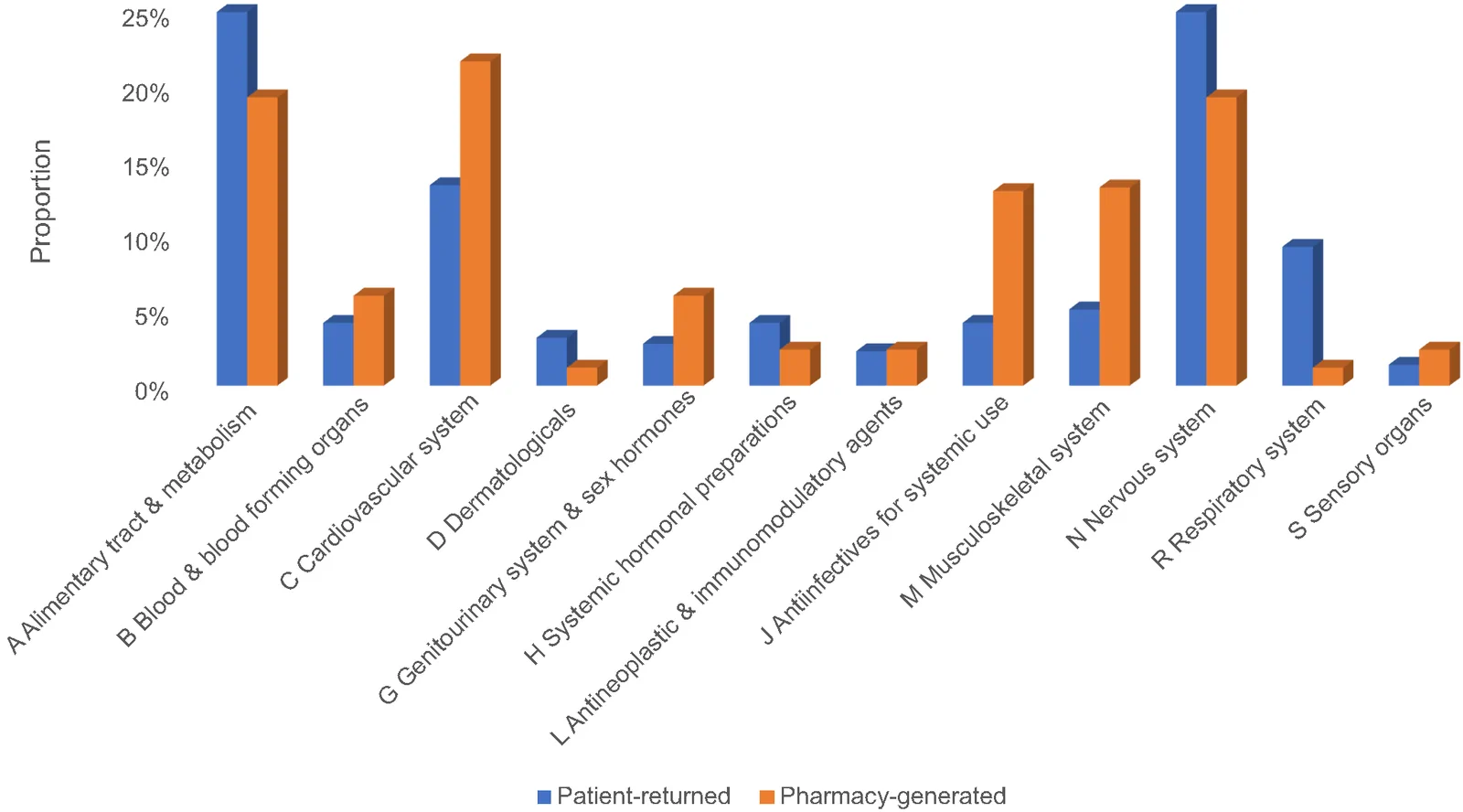
Medication waste by ATC Classification (% of Total) - patient-returned and pharmacy-generated

ATC first-level classes A, C and N accounted for the highest proportion of medicines waste, but proportions differed between the two sources (Fig. 1). For patient-returned medicines these were Class A - alimentary tract and metabolism (25%), Class N - nervous system (25%), and Class C - cardiovascular system (13.4%). For pharmacy-generated waste, the leading categories were Class C - cardiovascular system (21.7%), Class A - alimentary tract and metabolism (19.3%), and Class N - nervous system (19.3%). Anti-infectives in tablet form accounted for 4.2% of all patient returns.

### Reasons for wasted medicines from patient returns

**Fig. 2:**
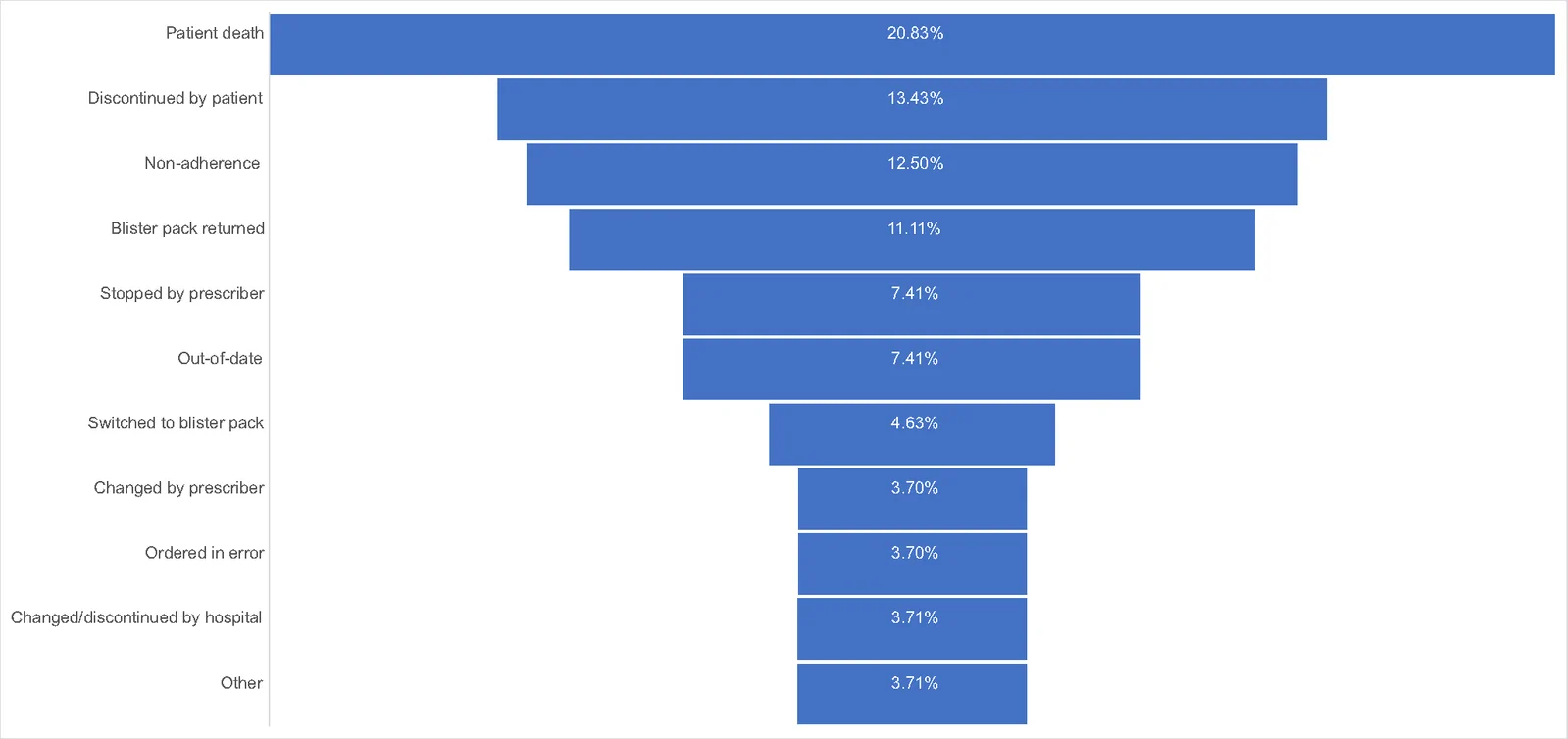
Reported reasons for medication returned by patients

The primary reasons for patient-returned were patient death (20.8%), treatment discontinued by the patient (13.4%), and general non-adherence resulting in medication accumulation (12.5%) (Fig. 2). Reasons for return were not recorded for 7.9% of items.

**Fig. 3:**
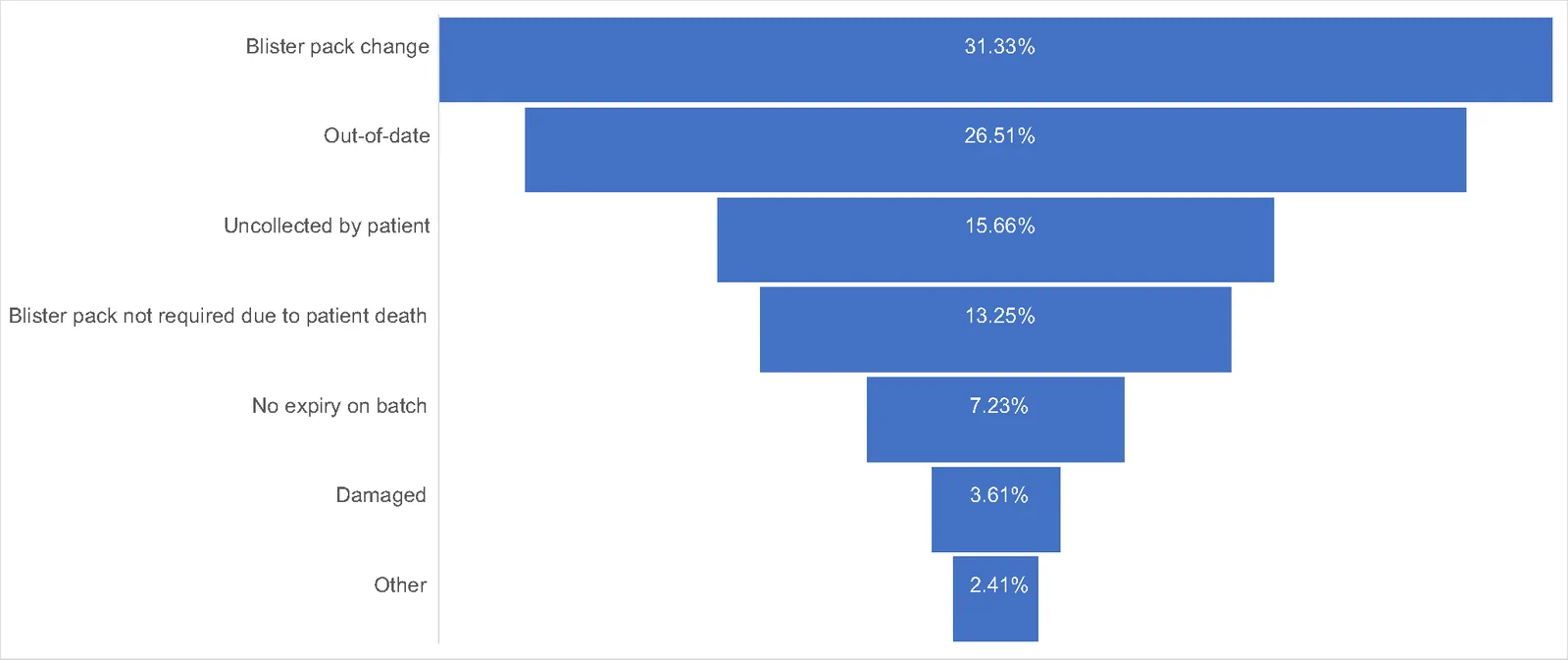
Reasons for pharmacy-generated waste medicines (% of total)

For pharmacy-generated waste, the most common reasons for disposal were blister pack amendments following treatment changes (31.3%), expired medicines (26.5%), and medicines left uncollected by patients (15.7%) (Fig. 3). Two pharmacy sites did not record any pharmacy-generated medication waste over the audit period. This was explained by both pharmacies having conducted extensive stock-checking and the destruction of any expired medicines in the recent period preceding the conduct of this audit.

### Economic assessment

The total estimated economic value of wasted medicines generated by patient returns and pharmacy-generated was €6,049.87 across the four pharmacies during the study period (Table 2). Patient-returned items accounted for €4,928.39 (81.5%) and pharmacy-generated waste for €1,121.48 (18.5%). The average cost per wasted item was €20.23. Prescription items represented the majority of the economic burden, with an estimated cost of €5,903.66 (97.6%), while OTC products accounted for €146.21 (2.4%).

**Table 2:** Quantity and estimated value of medicines waste – patient-returned and pharmacy-generated

Source		Number of items	% of total items	Total units	Total value (€)	% of total value
Patient-returned	216		72%	6451	4928.39	81.5%
Pharmacy-generated	83		28%	2035	1121.48	18.5%
Total	299		100%	8486	6049.87	100%

In terms of categories of medicines, the main contributors to costs were ATC class H - systemic hormonal preparations excluding sex hormones and insulins, at €2,005.59 (33.2%), ATC class N - nervous system, at €1,337.76 (22.1%), and ATC class A - alimentary tract and metabolism, at €616.48 (10.2%). Although solid oral dosage forms, including tablets and capsules, accounted for the majority of waste items (75%), they represented some 36% (€2,150.92) of the total estimated economic burden. In contrast, injectable solutions comprised 8.7% of items, but had a substantially higher per-item cost, contributing to 46.4% (€2,804.30) of the total cost.

The top ten medicines with the highest total estimated costs comprised both high-cost medicines and lower-cost medicines returned in larger quantities (Table 3). High-cost patient-returned items included High-Tech scheme medicines i.e. octreotide injection and the oral combination formulation, Teysuno^®^, as well as medicines intended for emergency use, such as adrenaline auto-injectors and buccal midazolam, returned unused following expiry. Within pharmacy-generated waste, one High-Tech scheme medicine (teriparatide) alone was recorded, accounting for 23.8% of the total cost of pharmacy-generated waste (Table 2).

**Table 3:** Top 10 Medicines driving the highest value – patient-returned & pharmacy-generated

Patient-returned		Pharmacy-generated	
Medicine	Total value (€)	Medicine	Total value (€)
Octreotide injection*	1705.60	Teriparatide*	267.04
Oxycodone	319.56	Levomepromazine	210.48
Teysuno^®^*^#^	308.54	Linagliptin	48.05
Adrenaline	246.50	Sodium valproate	41.4
Midazolam	218.58	Mirabegron	38.21
Famciclovir	178.65	Insulin Aspart	37.63
Trelegy^®^~	173.85	Fluconazole	35.72
Levonorgestrel	126.26	Leflunomide	35.04
Ondansetron	122.36	Atorvastatin/ezetimibe	33.62
Sertraline	115.04	Sacubitril/valsartan	33.54

*High Tech Scheme Medicine;^#^ Teysuno^®^: combination product tegafur, gimeracil and oteracil; ~Trelegy^®^: combination inhaler of fluticasone furoate, umeclidinium bromide, vilanterol

Based on this audit in four community pharmacies, the mean value of medication waste generated per pharmacy of €1,512.47 over a four-week period extrapolates to an estimated value per pharmacy of €19,662.11 annually (52 weeks). There were 1,908 community pharmacies registered in Ireland in November 2025 [45]. Applying the estimated annual value generated in this audit to all community pharmacies, the annualised average value of medicines waste per pharmacy equates to a national economic burden in the region of €37.52 million.

## Discussion

This audit provides an initial observational snapshot of medication waste generated over four weeks across four community pharmacies in Ireland and a hypothetical extrapolated national economic value. It identifies key trends in the types of medicines wasted, the primary reasons for waste, and the consequent economic implications.

Patient death was the most frequently reported reason for initiating a medicine return in the audit, which is consistent with previous studies [38, 39, 42, 46]. Many of the returned medicines were anticipatory end-of-life medicines that remained unused including octreotide, midazolam and levomepromazine. There is a fine balance to be considered between ensuring adequate end-of-life symptom control and avoiding oversupply and subsequent waste [47]. In Ireland, prescriptions for patients eligible for free medicines under the GMS (subject only to a limited prescription levy per item) who are terminally ill are often prescribed and dispensed large or monthly quantities, despite dosing often requiring escalation and adjustment every few days [48]. It should also be noted that the “patient death” category may capture medicines that accumulated for other reasons prior to death, such as poor adherence, therapy changes, or symptom changes.

A quarter of returned medications (26%) was either as a result of patients deciding to stop medications themselves or not adhering to their prescribed medication, thus exceeding the proportion of medications returned due to the death of a patient. However, it is difficult to postulate the many and varied reasons for this, as patients may discontinue taking some medicines for a variety of secondary reasons, such as perceived lack of benefit or side effects, which were not captured in this audit. Non-adherence is a well-recognised global issue, with adherence among individuals with chronic conditions estimated at 50% [47, 49]. This may have significant consequences with the potential for sub-optimal disease management, increased hospitalisations, and greater healthcare resource utilisation [29, 50]. Cardiovascular medicines are primarily prescribed for chronic conditions, yet adherence is often sub-optimal due to limited perceived benefit and adverse effects [50–52]. A recent study conducted in the Netherlands also reported that cardiovascular medicines represented the highest unused medication in a survey of community pharmacies [37]. Incomplete adherence to antibiotic regimens is also of concern, as it may reduce treatment effectiveness and contribute to antimicrobial resistance [53, 54]. In our audit, 4.2% of patient returns were classified as ‘anti-infectives for systemic use’, similar to the rate returned in an antibiotic amnesty in the UK recently [55], with flucloxacillin accounting for the highest number of units returned. Potential factors influencing antibiotic non-adherence include early symptom resolution and complex dosing schedules, such as the four-times-daily regimen required for flucloxacillin [56]. Given their critical role in preventing adverse health outcomes, improving adherence to medicines is essential to maximise therapeutic benefit and minimise medication waste.

It was notable that no liquid antibiotics were returned, despite these formulations rarely being consumed in full due to fixed pack sizes, frequent dosing regimens and some element of poor palatability among paediatric patients [57, 58]. This suggests possible inappropriate disposal by patients, such as discarding into household waste or disposal down sinks, which poses environmental risks and may contribute to antimicrobial resistance [20]. Topical and liquid formulations are particularly prone to inappropriate disposal [32]. Inadequate or an absence of counselling on the safe disposal of residual medicines is likely a contributing factor. A study by Lim et al. reported that over 59% of Australian pharmacists recommended unsatisfactory disposal methods for liquid antibiotics [59]. Although pharmacists are well positioned to provide disposal guidance, evidence suggests many lack awareness of recommended practices themselves, potentially exacerbated by the absence of robust guidance resulting in variations in procedures [60].

Medicines classified under the ‘alimentary tract and metabolism’ (25%), ‘nervous system’ (25%), ‘cardiovascular system’ (13.4%) and ‘respiratory system’ (9.3%) ATC categories accounted for the largest proportion of patient-returned medication waste. Similar findings have been reported in other studies, although the ranking of categories varies across settings [26, 38, 40–42, 61]. Medicines within the ‘nervous system’ and ‘alimentary tract and metabolism’ categories may be more frequently prescribed on an as-required basis for symptomatic relief. As-required medicines are a frequent source of waste in many studies [40–43]. Oversupply of these items may contribute to accumulation of unused medicines in the home which is unsafe and can lead to harm or misuse [62]. Globally, the overall rate of prescriptions is increasing due to aging populations with multi-morbidities resulting in polypharmacy approaches for chronic disease management, and this trend is set to continue [6]. Overprescribing of medicines can contribute to medication waste, compounded by non-adherence as evidenced in this audit. In addition, prescriptions that are filled but not consumed contribute to a significant carbon footprint along a medicines lifecycle without any therapeutic benefit [63]. From the patient perspective, in tandem with everyday life, increased consumerism has become a feature of healthcare with a widespread belief that many of life’s challenges can be treated or addressed medically [64]. As opined over two decades ago by Moynihan and Smith, there is almost certainly ‘too much medicine’ [65].

**Fig. 4:**
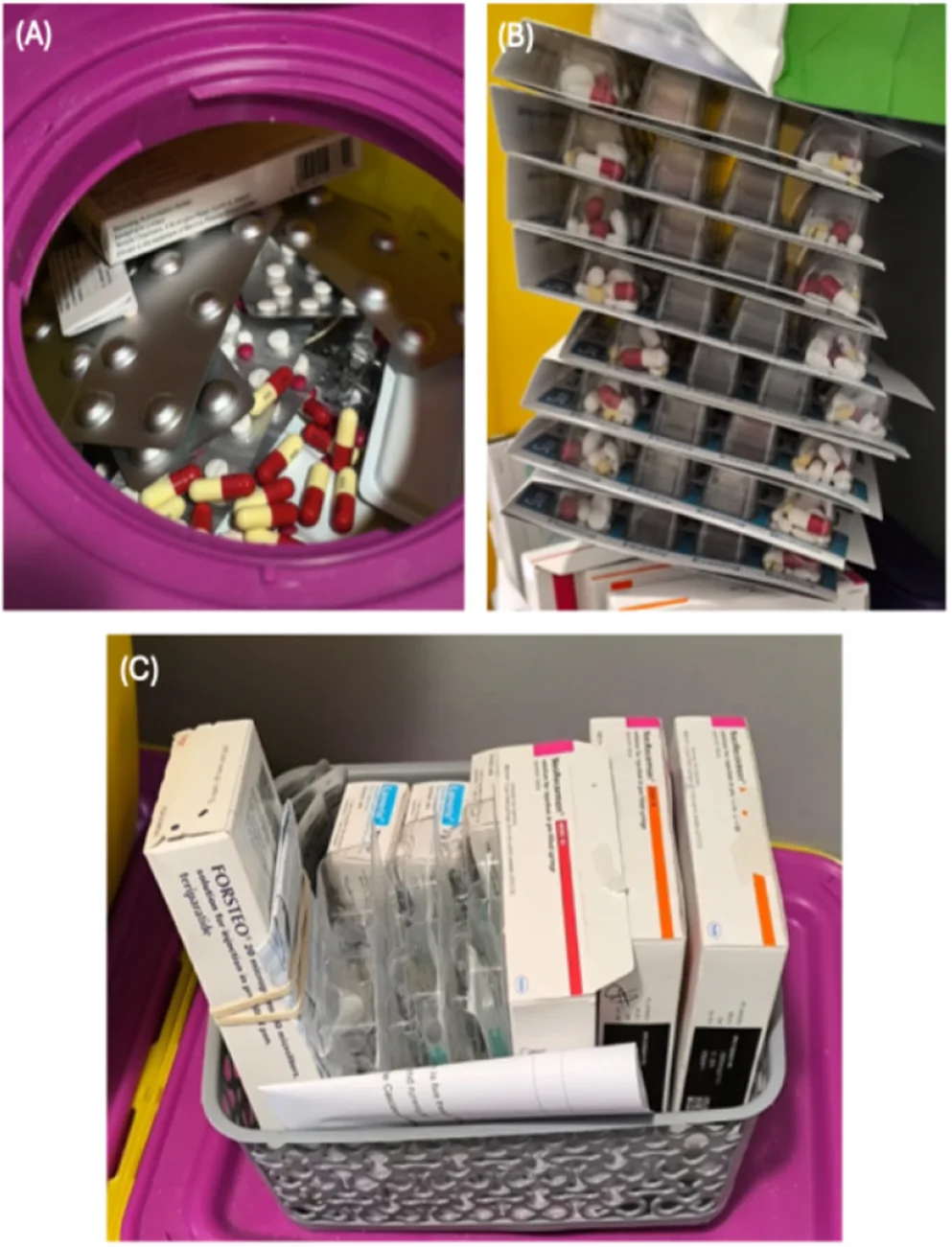
Visual examples of medication waste collected during the audit period - (**A**) Contents of a medicines’ disposal bin containing intact blister packs, boxed medicines and a paper patient information leaflet. (**B**) Collection of blister packs awaiting disposal. (**C**) Basket of expired High-Tech scheme medicines designated for disposal

For pharmacy-generated waste, the most frequently documented cause was blister pack amendments. Although medication compliance aids (MCAs) are intended to support adherence, evidence of their effectiveness remains limited, and their preparation is both labour- and resource-intensive [29]. The need to discard blister packs after treatment changes represents a significant source of preventable waste (Fig. 4). One participating pharmacy attempted to reduce this by modifying existing packs rather than discarding them entirely; however, substantial waste was still generated. This highlights the need to critically re-evaluate the routine use of MCA devices in practice.

Medicines expiring in pharmacies before they are dispensed was the second most common cause of pharmacy-generated waste. Uncollected prescriptions accounted for 15.7% of all pharmacy-generated medication waste. This was likely driven by the routine practice of preparing prescriptions prior to patient request, frequently prompted by a prescription transmitted through Healthmail, (a secure electronic mail system where prescriptions are transmitted direct from prescribers to a designated pharmacy) which was observed in three of the four participating pharmacies. While uncollected medicines that remain within the pharmacy can generally be reused, this is not possible when expiry dates or batch numbers are unidentifiable or the product has become too short-dated to re-enter pharmacy stock, resulting in avoidable waste.

Waste prevention is at the heart of addressing the problems associated with the generation of medication waste and aligns with the Sustainable Development Goals [37]. Reducing medication waste requires the engagement of multiple stakeholders, including prescribers, pharmacists, patients, and policymakers underpinned by rigorous implementable legislation [35]. The EU has a broad range of legislation under the auspices of the EU Green Deal focussing on environmental issues, including waste management [66]. Proposed revisions to EU pharmaceutical legislation, prioritising stronger environmental protection through improved enforcement of existing requirements to address the ecological impact of medicines may assist in addressing the problem [67]. Despite the importance of medicine waste collection, implementation varies significantly across Europe and globally with some countries adopting extended producer responsibility (EPR) schemes, placing greater economic and organisational responsibility for household medicines disposal on the pharmaceutical industry [68].

Responsible disposal of medicines is predicated upon the availability of nationally funded take back schemes, as not all waste is preventable. Therefore, proper collection and safe disposal of unused medicines are essential to limit environmental contamination, although some countries do not provide such schemes including Ireland [31, 32, 69]. The proposed funded scheme announced in 2025 in Ireland, will have multiple benefits including reduced risk from unused medicines stored in the home, inappropriate disposal with recognised environmental implications and reducing the financial burden on community pharmacies, although there is an absence of detail at present (April 2026) regarding the precise waste streams to be included in the scheme [36].

There are several strategies to encourage reductions in overprescribing in the clinical setting by prioritising prevention, switching to alternative non-pharmacological interventions including social prescribing, reviewing the provision of as required and repeat prescriptions on a regular basis, and reducing antibiotic prescribing [63, 64, 70–72]. There are several resources available to guide such initiatives including the ‘Choosing Wisely’ initiative with demonstrable evidence of reducing the level of low-value care provision [73]. Pharmacists can play an active role in encouraging and promoting patient adherence and enhancing self-care strategies, a key tenet of sustainable healthcare. Medication use reviews (MURs) offer a structured, patient-centred approach to improving adherence and reducing waste, although a formal MURs initiative is not available in Ireland [74]. However, the success of the recent EU-funded iSYMPATHY initiative in addressing polypharmacy and adherence across three jurisdictions, including Ireland, demonstrated the potential for wider implementation nationally [75]. Embedding deprescribing and medicines optimisation strategies into the routine functions of prescribing and supplying of medicines respectively would reduce the potential for the subsequent generation of medication waste [36, 76]. Supporting community pharmacy-led antimicrobial stewardship initiatives would allow oversight of antimicrobial use, which could be extended to innovative programmes to train children to take tablets rather than liquids, to reduce wasted liquid antibiotics [77–80]. Patients should also be included in any waste reduction strategies through engagement in public awareness campaigns, with existing evidence of success [81].

Although pharmacy-generated medication waste represented just over a quarter of the medication waste identified in this audit, there are still opportunities to implement waste-saving measures. Changes to patient medication prompting the need to change prepared blister packs was responsible for a third of this waste. Many of these changes may have been unavoidable but processes to prevent the need for disposal could be achieved by selecting patients on dose escalation regimens, or whom have suffered from acute exacerbations, or indeed those receiving palliative care. The use of original packs of medicines should be the preferred choice for the supply of medicines in the absence of a specific need for an MCA in all settings [82]. MCAs should be offered primarily to support patient-safety and adherence, rather than patient preference or convenience. Changes to how pharmacies are likely to charge their patients for the provision of blister packs during the course of 2026 is likely to result in a reduction in the number of medicines supplied in this format by pharmacies [83]. Uncollected prescriptions accounted for 16% of pharmacy-generated waste which prompts the need to review processes around repeat dispensing generated via the Healthmail system without prior confirmation by patients. The recent Royal Pharmaceutical Greener Pharmacy Guide highlighted repeat dispensing as an opportunity to prevent avoidable medicines waste generation [84]. Therefore, the introduction of interventions or measures requiring patients to confirm the necessity of each item on their repeat prescriptions at the point of it being ordered or supplied may be merited. Such practices have been shown to be successful in the UK, in reducing waste and furthermore, helping to identify non-adherence and adverse drug reactions with ‘Only order what you need’ campaigns now embedded across many UK primary care trusts [85].

Optimising stock control processes within pharmacies to prevent drug expiry will also benefit waste avoidance. Innovative initiatives, such as PharmaSwap in the Netherlands, demonstrate the feasibility of the redistribution of medicines coming close to expiry between pharmacies [86]. The practical challenges required to support PharmaSwap include cold chain management and the additional GHG emissions associated with transport [27]. However, these concerns could be mitigated by utilising existing delivery systems, with wholesalers and distributors facilitating the exchange of medicines [87].

The potential economic impact of medication waste is considerable. In 2010, it was estimated that £300 million was wasted annually in primary care in the UK, while in 2023, a figure of £18 million annually was estimated for Northern Ireland [29, 88]. This study has estimated the average value of waste medicines generated per pharmacy over the audit period was €1,512.47, which when annualised and extrapolated to all pharmacies in Ireland, exceeds €37.5 million. While it is acknowledged that this estimate is based on a small, short audit limited to four pharmacies and may be associated with significant uncertainty, it provides a useful context on which to base the potential extent and economic impact of wasted medicines in Ireland. It also provides an indication of the healthcare resources lost to medication waste that could otherwise be redirected to patient care, should tailored waste reduction strategies be introduced.

In recognition of the inappropriate disposal of unused or expired household medicines in many EU countries, an international symposium on ‘Minimising the Ecological and Economic Burden of Medication Waste in Europe’ involving experts from 15 of the 27 EU countries was convened in Brussels in June 2025 [89]. To address the challenges, the symposium culminated in the adoption of the Brussels Declaration on the Responsible Disposal of Unused and Expired Medications in Europe [90]. The Declaration calls for immediate, coordinated Europe-wide action to close policy gaps, empower citizens, and strengthen the sustainability of European healthcare systems. This recommends a three-pronged strategy to address the problem i.e. (a) coordinated awareness campaigns targeting patients and citizens, (b) clear and standardised graphical symbols on medicine packaging and (c) national information platforms for safe disposal guidance. Among the innovative strategies discussed was the potential re-use of returned medicines, but there are significant challenges to its implementation [87, 91, 92].

## Limitations

While this audit provides valuable initial insights into the volume, nature, and drivers of medication waste in community pharmacy practice, several limitations should be considered. Data collection occurred over a relatively short period, which may not capture seasonal variations in prescribing patterns, patient behaviours, or waste generation. The audit was also confined to four suburban pharmacies, potentially limiting generalisability to other geographic settings with different dispensing volumes and patient demographics. The value of medicines waste may be overestimated by the use of list prices that do not include discount rates negotiated by pharmacies for the purchase of those medicines.

## Conclusion

The audit results show that medications returned by patients accounted for the greatest proportion of waste, both in volume and cost. Waste generated within pharmacies was primarily linked to prescription changes requiring blister pack alterations and to expired stock. When extrapolated nationally, the financial impact of this wasted medication appears substantial. These findings highlight the need to implement the proposed national scheme to ensure the safe disposal of unavoidable waste, supported by strategies aligned with those outlined in the Brussels Declaration. Further research is needed to more comprehensively assess medicines waste in Ireland, drawing on a larger and more diverse sample of community pharmacies to better understand its scale and underlying causes. Similar research is also required in the hospital sector to assess medicines waste there. Such evidence would inform the development of targeted prevention strategies for key stakeholders across the medicines’ lifecycle, without compromising patient safety or quality of care. Adopting a more sustainable framework for medicines use would not only reduce environmental harm, but also enhance resource efficiency and improve patient care. Human health and environmental health are deeply interconnected, and coordinated action is essential to safeguard both.

## Data Availability

The datasets generated and/or analysed during the current study are available from the corresponding author on reasonable request.
